# Spectral clustering for TRUS images

**DOI:** 10.1186/1475-925X-6-10

**Published:** 2007-03-15

**Authors:** Samar S Mohamed, Magdy MA Salama

**Affiliations:** 1 E&CE Dept., University of Waterloo, Waterloo, Ontario, Canada

## Abstract

**Background:**

Identifying the location and the volume of the prostate is important for ultrasound-guided prostate brachytherapy. Prostate volume is also important for prostate cancer diagnosis. Manual outlining of the prostate border is able to determine the prostate volume accurately, however, it is time consuming and tedious. Therefore, a number of investigations have been devoted to designing algorithms that are suitable for segmenting the prostate boundary in ultrasound images. The most popular method is the deformable model (snakes), a method that involves designing an energy function and then optimizing this function. The snakes algorithm usually requires either an initial contour or some points on the prostate boundary to be estimated close enough to the original boundary which is considered a drawback to this powerful method.

**Methods:**

The proposed spectral clustering segmentation algorithm is built on a totally different foundation that doesn't involve any function design or optimization. It also doesn't need any contour or any points on the boundary to be estimated. The proposed algorithm depends mainly on graph theory techniques.

**Results:**

Spectral clustering is used in this paper for both prostate gland segmentation from the background and internal gland segmentation. The obtained segmented images were compared to the expert radiologist segmented images. The proposed algorithm obtained excellent gland segmentation results with 93% average overlap areas. It is also able to internally segment the gland where the segmentation showed consistency with the cancerous regions identified by the expert radiologist.

**Conclusion:**

The proposed spectral clustering segmentation algorithm obtained fast excellent estimates that can give rough prostate volume and location as well as internal gland segmentation without any user interaction.

## 1. Background

Prostate disease has a foremost impact on the quality of life of elderly men. Benign enlargement of the prostate frequently causes bladder outlet difficulty [1,2]. Malignant diseases of the prostate are considered a significant cause of death. Moreover, there has been an increase in the reported prevalence of prostate cancer. This is believed to be due to increased prostate awareness, prostate specific antigen (PSA) screening, and imaging techniques such as TransRectal UltraSound (TRUS).

Prostate Cancer is typically diagnosed by conduction a biopsy operation. Prostate Cancer diagnosis is usually aided by using TRUS images. This procedure is two manifold, first the prostate boundary is detected, Second the prostate tissue should be segmented and/or classified to different regions. The work done in this paper focuses on the TRUS image segmentation. The gland is first segmented from the background, second the outlined gland is further segmented into different regions.

The first segmentation process (prostate boundary segmentation from TRUS images) is crucial for some major applications in prostate disease decisions such as aiding diagnosis [3,4] and treatment planning [5,6]. Prostate Brachtherapy involves accurately insertion of radioactive materials (seeds) into the gland according to a predetermined plan [5] in which the gland location and boundary should be determined.

The purpose of the second segmentation process is mainly Computer Aided Diagnosis (CAD). The internal segmentation process involves segmenting the prostate gland into regions where different regions that represent different tissue textures in the gland are identified. The region segmentation highlights the different gland regions for either the purpose of feature analysis, or to augment the radiologist decision in highlighting the suspicious regions.

The internal segmentation system proposed in this paper can serve as a preprocessing stage for any CAD system. In the earlier work features were constructed for the whole image, where the whole image is divided into small squares and features are then constructed from each of those squares [7,8]. This is considered a time consuming process. Therefore internally segmenting the prostate and studying only the highly suspicious regions is expected to be more accurate and efficient.

The proposed segmentation method has a different foundation than the previously proposed segmentation systems as it relies mainly on graph theory techniques. On the other hand, the older segmentation methods are mainly: edge base segmentation, texture based segmentation and model based segmentation. Each of these methods requires prior knowledge such as a seed point or three points on the boundary. In the proposed TRUS image segmentation method, spectral clustering treats the image as a weighted undirected graph and finds the segmentation result by obtaining the minimum cut of this weighted graph based on the graph theory methods without any prior knowledge to either a seed point or any point on the boundary.

The TRUS images used in this work are obtained from University of Western Ontario and are derived from Aloka 2000 ultrasound machine using a broadband 7 MHz linear transducer and a field of view of approximately 6 cm. A set of 29 radiologist identified TRUS images were used for this study.

## 2. Related work

The prostate boundaries are typically identified from TRUS images. Although manual outlining of the prostate border enables the prostate volume to be determined accurately [9], it is time consuming and tiresome. Moreover, since, in TRUS images' quality is not very good, therefore, traditional edge detectors are unable to extract the correct boundaries. Therefore, a number of investigations have been devoted to design automatic or semi-automatic methods that are suitable for segmenting the prostate boundary from ultrasound images.

A segmentation method that depends on clustering each pixel of an ultrasound image was introduced in [10]. In this method, along with the relative position of the pixel, four energy measures were used to determine the cluster that the pixel belongs to. A drawback of this method is that the number of clusters is not predictable for a particular image; therefore, the prostate might be represented by disconnected regions.

Artificial Neural Networks (ANNs) was also introduced as a method for prostate segmentation in transrectal ultrasound images [11]. This method segments images to prostate and non-prostate regions. Three neural network architectures have been proposed. This method needs lots of training data in order to train the ANN; moreover the training process is lengthy and computationally expensive.

Active contours were introduced in [12] and are used since then as one of the main methods for prostate boundary detection. The active contours model is used in [13] for prostate boundary detection where constraints were imposed on the model's deformation according to a predefined model shape. In this method, one-dimensional wavelet transform was applied on the radial function of both the prior model and the deformed model. While it was demonstrated that this method detected the prostate boundary accurately for typical gland shapes, the dependence of the statistically derived prior model has limited its ability to segment the prostate with atypical shape.

In an attempt to enhance the active contours method a cubic spline interpolation technique was used in [14] to identify an initial contour based on four user-defined points. Then, Lobregt's discrete dynamic contour (DDC) model [15] was used to refine the boundary. This method was shown to be effective if the initial contour was defined accurately, however, the result was less satisfactory for segmenting an irregular boundary that could not be accurately approximated by the initial contour, and further human intervention was required under this condition.

Another semi-automatic segmentation algorithm based on the dyadic wavelet transform and the discrete dynamic contours was used in [16]. In this method first a spline interpolation is used to determine the initial contour based on four user-defined initial points. Then the discrete dynamic contour refines the initial contour based on the approximate coefficients and the wavelet coefficients generated using the dyadic wavelet transform. A selection rule is used as well to choose the best contour.

A common deformable model was also used in [17] to segment the prostate in transrectal ultrasound images. The new enhancement was the use of a Gabor filter bank in both multiple scales and multiple orientations to characterize the prostate boundaries. The Gabor features are then reconstructed to be invariant to the rotation of the ultrasound probe. Then, the segmentation is obtained by minimizing the energy function of the prostate shape model. The model focuses on the similarity of different Gabor features at different deformation stages using a multiresolution technique.

Another deformable models based research presented an approach where model initialization and constraining model evolution are based on prior knowledge about the prostate shape [18]. The prostate shape has been modeled using deformable superellipses.

Deformable models were also used in [19] for automatic segmentation of trans-abdominal ultrasound images of the prostate. In this method a filter is used to enhance the contours without changing the information in the image. Adaptive morphological and median filtering are employed to detect the noise-containing regions and smooth these areas. Then a heuristic optimization algorithm begins to search for the contour initialized from a prostate model.

All the active contours based methods depend mainly on the initial points set by the user as well as the initial contour generation. Most of the research is focused on changing the number of initial points or changing the method used to obtain the initial contour. Optimizing the energy function is also an area of research using the active contour method.

Another segmentation technique to extract prostate contours from Transrectal Ultrasound images using Sticks filter to reduce the speckle was proposed in [21]. Equi-spaced radii were then projected from an arbitrary seed point inside the prostate cavity towards its boundary. Candidate edge points are then obtained along each radius which include the edge points and some false points. This approach is dependent on the choice of the seed point which might mislead the prostate contour extraction method.

The sticks method was used in another algorithm for prostate boundary detection in [20]. The algorithm provides prostate edge detection as a visual guide to the observer using edge delineation. It is then followed by manual editing. This edge detection algorithm contains three stages. First, the sticks algorithm is used to enhance contrast and reduce speckle in the image. Second, the resulting image is smoothed using an anisotropic diffusion filter. Finally, some basic prior knowledge of the prostate, such as shape and echo pattern, is used to detect the most probable edges which indicate the prostate shape. In the last stage, the information is then integrated by using a manual linking procedure on the detected edges. The drawback of this method is that it depends on prior knowledge of some prostate features, a limitation that makes it limited to typical prostate gland shapes and echo patterns. Moreover it needs manual editing at the final stage to obtain the prostate boundaries.

Most of the above-mentioned methods depended on statistical estimation for initialization and some of these initialization methods were not accurate enough [17]. Some other methods depend on choosing the right seed point, otherwise the algorithm will not converge to the right boundary. While all the above methods depend on solving optimization problems that are parameter sensitive and time consuming.

Generally, prostate segmentation methods have limitations when the image contains shadows with similar gray level and texture attached to the prostate. In these cases the segmentation error may increase considerably. Another problem may be the lack of sufficient number of training images if a learning technique is used. Algorithms based on active contours have been quite successfully implemented with the major drawback that they depend on user interaction to determine the seed points or the initial snake.

Based on the previous literature review of the existing methods, a new approach should ideally be:

• Independent on user interaction as user interaction (e.g. defining seed points or initial contours) has drawbacks such as time consumption, human error etc.

• Independent on training images where training images is typically difficult to obtain, especially if the samples should be prepared by an expert. Hence, sample-based learning should be avoided.

• Independent on noise level where the approach must be robust with respect to the presence of noise and shadow.

The proposed segmentation algorithm in this paper has a totally different basis as it doesn't depend on the inherited snakes' algorithm. This will get rid of designing the energy function, optimizing it and accurately selecting the seed points or initial contour points.

## 3. Spectral clustering

The Human Visual System (HVS) can effectively identify objects in a scene and can often segment the scene to coherent segments or clusters. There has been a tremendous amount of research done to achieve the same level of performance obtained by the HVS. Various methods have been introduced in literature to segment ultrasound images such as Kalman filters [21] and statistical shape models in which the prostate is segmented from the background in order to determine the gland volume. However most of these methods require large amount of human interaction. The Gabor filters were introduced in this field as a method to internally segment the prostate gland that was already manually segmented from the background [22] in which a Gabor filter was designed to automatically and accurately segment the TRUS images.

Spectral Clustering methods are applied in this paper in order to give a fast segmentation results that don't require either filter design or any human interaction. Spectral Clustering have been introduced for data clustering and was applied in different fields. The spectral clustering usually represents the data by a weighted graph and the eigenvectors of the affinity (or similarity) matrix of this graph are used for the segmentation [23]. In the problem of image segmentation the image pixels are considered as the data points as shown in [24].

### 3.1. Graph based image segmentation

Given an image I, a graph *G *= (*V*, *E*, *W*) is constructed with the pixels represented by the graph nodes *V*, and the pixels within a distance *V *≤ *G*_*r *_are connected by a graph edge *E*. The weight *W*(*i*, *j*) measures the likelihood of pixel *i *and *j *being in the same cluster. Partitioning of this graph represents the image segmentation [24-26].

#### Assigning weights to graph edges

The pair-wise pixel affinity graph determines the segmentation accuracy. Therefore, as recommended in [25] two simple local grouping cues are used which are the intensity and contours.

**1. Grey Level Intensity: **neighboring pixels with close intensity are most likely to be in the same region.

Wi(i,j)=e−‖Xi−Xj‖2σx−‖Ii−Ij‖2σI     (1)
 MathType@MTEF@5@5@+=feaafiart1ev1aaatCvAUfKttLearuWrP9MDH5MBPbIqV92AaeXatLxBI9gBaebbnrfifHhDYfgasaacH8akY=wiFfYdH8Gipec8Eeeu0xXdbba9frFj0=OqFfea0dXdd9vqai=hGuQ8kuc9pgc9s8qqaq=dirpe0xb9q8qiLsFr0=vr0=vr0dc8meaabaqaciaacaGaaeqabaqabeGadaaakeaacqWGxbWvdaWgaaWcbaGaemyAaKgabeaakiabcIcaOiabdMgaPjabcYcaSiabdQgaQjabcMcaPiabg2da9iabdwgaLnaaCaaaleqabaGaeyOeI0YaaSaaaeaadaqbdaqaaiabdIfaynaaBaaameaacqWGPbqAaeqaaSGaeyOeI0IaemiwaG1aaSbaaWqaaiabdQgaQbqabaaaliaawMa7caGLkWoadaahaaadbeqaaiabikdaYaaaaSqaaGGaciab=n8aZnaaBaaameaacqWG4baEaeqaaaaaliabgkHiTmaalaaabaWaauWaaeaacqWGjbqsdaWgaaadbaGaemyAaKgabeaaliabgkHiTiabdMeajnaaBaaameaacqWGQbGAaeqaaaWccaGLjWUaayPcSdWaaWbaaWqabeaacqaIYaGmaaaaleaacqWFdpWCdaWgaaadbaGaemysaKeabeaaaaaaaOGaaCzcaiaaxMaadaqadaqaaiabigdaXaGaayjkaiaawMcaaaaa@58FC@

Where *X*_*i *_and *I*_*i *_represent pixel location and intensity.

Connecting pixels considering only intensity and location usually gives bad segmentation due to the texture that is present in the TRUS images. Therefore the principal image contours (edges) are also considered for the segmentation of TRUS images.

**2. Dominant Contours: **the image edges are considered useful when the neighboring regions have the same cutter. The affinity between two pixels is calculated by measuring the image edges between them.

WC(i,j)=e−max⁡x∈line(i,j)‖Edge(x)‖2/σc     (2)
 MathType@MTEF@5@5@+=feaafiart1ev1aaatCvAUfKttLearuWrP9MDH5MBPbIqV92AaeXatLxBI9gBaebbnrfifHhDYfgasaacH8akY=wiFfYdH8Gipec8Eeeu0xXdbba9frFj0=OqFfea0dXdd9vqai=hGuQ8kuc9pgc9s8qqaq=dirpe0xb9q8qiLsFr0=vr0=vr0dc8meaabaqaciaacaGaaeqabaqabeGadaaakeaacqWGxbWvdaWgaaWcbaGaem4qameabeaakiabcIcaOiabdMgaPjabcYcaSiabdQgaQjabcMcaPiabg2da9iabdwgaLnaaCaaaleqabaGaeyOeI0IagiyBa0MaeiyyaeMaeiiEaG3aaSbaaWqaaiabdIha4jabgIGiolabdYgaSjabdMgaPjabd6gaUjabdwgaLjabcIcaOiabdMgaPjabcYcaSiabdQgaQjabcMcaPaqabaWcdaqbdaqaaiabdweafjabdsgaKjabdEgaNjabdwgaLjabcIcaOiabdIha4jabcMcaPaGaayzcSlaawQa7amaaCaaameqabaGaeGOmaidaaSGaei4la8ccciGae83Wdm3aaSbaaWqaaiabdogaJbqabaaaaOGaaCzcaiaaxMaadaqadaqaaiabikdaYaGaayjkaiaawMcaaaaa@5E7E@

Where *line*(*i*, *j*) is a straight line joining pixels *i *and *j *and *Edge*(*x*) is the edge strength at location(*x*).

The two cues are combined in this work in the form:

WI(i,j)×WC(i,j)+αWC(i,j)     (3)
 MathType@MTEF@5@5@+=feaafiart1ev1aaatCvAUfKttLearuWrP9MDH5MBPbIqV92AaeXatLxBI9gBaebbnrfifHhDYfgasaacH8akY=wiFfYdH8Gipec8Eeeu0xXdbba9frFj0=OqFfea0dXdd9vqai=hGuQ8kuc9pgc9s8qqaq=dirpe0xb9q8qiLsFr0=vr0=vr0dc8meaabaqaciaacaGaaeqabaqabeGadaaakeaadaGcaaqaaiabdEfaxnaaBaaaleaacqWGjbqsaeqaaOGaeiikaGIaemyAaKMaeiilaWIaemOAaOMaeiykaKIaey41aqRaem4vaC1aaSbaaSqaaiabdoeadbqabaGccqGGOaakcqWGPbqAcqGGSaalcqWGQbGAcqGGPaqkaSqabaGccqGHRaWkiiGacqWFXoqycqWGxbWvdaWgaaWcbaGaem4qameabeaakiabcIcaOiabdMgaPjabcYcaSiabdQgaQjabcMcaPiaaxMaacaWLjaWaaeWaaeaacqaIZaWmaiaawIcacaGLPaaaaaa@4C8F@

#### Spectral clustering segmentation algorithms

In [26], a clustering algorithm based on thresholding the largest eigenvector of the affinity matrix was suggested. While in [27] the authors have argued for using a totally different eigenvector for solving these types of segmentation problems. Rather than examining the first eigenvector of *W *they examined the generalized eigenvectors. Let *D *be the degree matrix of *W*:

D(i,i)=∑jW(i,j)     (4)
 MathType@MTEF@5@5@+=feaafiart1ev1aaatCvAUfKttLearuWrP9MDH5MBPbIqV92AaeXatLxBI9gBaebbnrfifHhDYfgasaacH8akY=wiFfYdH8Gipec8Eeeu0xXdbba9frFj0=OqFfea0dXdd9vqai=hGuQ8kuc9pgc9s8qqaq=dirpe0xb9q8qiLsFr0=vr0=vr0dc8meaabaqaciaacaGaaeqabaqabeGadaaakeaacqWGebarcqGGOaakcqWGPbqAcqGGSaalcqWGPbqAcqGGPaqkcqGH9aqpdaaeqbqaaiabdEfaxjabcIcaOiabdMgaPjabcYcaSiabdQgaQjabcMcaPaWcbaGaemOAaOgabeqdcqGHris5aOGaaCzcaiaaxMaadaqadaqaaiabisda0aGaayjkaiaawMcaaaaa@41D9@

The generalized eigenvector *y*_*i *_is a solution to:

*(D — W)y_i_ = λ_i_Dy_i_*     (5) 

Solving the generalized eigenvector minimizes the Ncut which in turn produce the optimum segmentation as proved in [24]. In this case the generalized eigenvector corresponding to the second smallest eigenvalue was used. Thresholding this second eigenvector to obtain the segmentation result was suggested. This method is adopted for the application of TRUS image segmentation and it yields to a segmentation that minimizes the normalized cut:

Ncut(A,B)=cut(A,B)asso(A,V)+cut(A,B)asso(B,V)     (6)
 MathType@MTEF@5@5@+=feaafiart1ev1aaatCvAUfKttLearuWrP9MDH5MBPbIqV92AaeXatLxBI9gBaebbnrfifHhDYfgasaacH8akY=wiFfYdH8Gipec8Eeeu0xXdbba9frFj0=OqFfea0dXdd9vqai=hGuQ8kuc9pgc9s8qqaq=dirpe0xb9q8qiLsFr0=vr0=vr0dc8meaabaqaciaacaGaaeqabaqabeGadaaakeaacqWGobGtcqWGJbWycqWG1bqDcqWG0baDcqGGOaakcqWGbbqqcqGGSaalcqWGcbGqcqGGPaqkcqGH9aqpdaWcaaqaaiabdogaJjabdwha1jabdsha0jabcIcaOiabdgeabjabcYcaSiabdkeacjabcMcaPaqaaiabdggaHjabdohaZjabdohaZjabd+gaVjabcIcaOiabdgeabjabcYcaSiabdAfawjabcMcaPaaacqGHRaWkdaWcaaqaaiabdogaJjabdwha1jabdsha0jabcIcaOiabdgeabjabcYcaSiabdkeacjabcMcaPaqaaiabdggaHjabdohaZjabdohaZjabd+gaVjabcIcaOiabdkeacjabcYcaSiabdAfawjabcMcaPaaacaWLjaGaaCzcamaabmaabaGaeGOnaydacaGLOaGaayzkaaaaaa@62FD@

where: *A *∪ *B *= *V and A *∩ *B *= 0, cut(A,B)=∑i∈A,j∈BW(i,j)
 MathType@MTEF@5@5@+=feaafiart1ev1aaatCvAUfKttLearuWrP9MDH5MBPbIqV92AaeXatLxBI9gBaebbnrfifHhDYfgasaacH8akY=wiFfYdH8Gipec8Eeeu0xXdbba9frFj0=OqFfea0dXdd9vqai=hGuQ8kuc9pgc9s8qqaq=dirpe0xb9q8qiLsFr0=vr0=vr0dc8meaabaqaciaacaGaaeqabaqabeGadaaakeaacqWGJbWycqWG1bqDcqWG0baDcqGGOaakcqWGbbqqcqGGSaalcqWGcbGqcqGGPaqkcqGH9aqpdaaeqbqaaiabdEfaxjabcIcaOiabdMgaPjabcYcaSiabdQgaQjabcMcaPaWcbaGaemyAaKMaeyicI4SaemyqaeKaeiilaWIaemOAaOMaeyicI4SaemOqaieabeqdcqGHris5aaaa@47EB@ and asso(A,V)=∑j∑i∈AW(i,j)
 MathType@MTEF@5@5@+=feaafiart1ev1aaatCvAUfKttLearuWrP9MDH5MBPbIqV92AaeXatLxBI9gBaebbnrfifHhDYfgasaacH8akY=wiFfYdH8Gipec8Eeeu0xXdbba9frFj0=OqFfea0dXdd9vqai=hGuQ8kuc9pgc9s8qqaq=dirpe0xb9q8qiLsFr0=vr0=vr0dc8meaabaqaciaacaGaaeqabaqabeGadaaakeaacqWGHbqycqWGZbWCcqWGZbWCcqWGVbWBcqGGOaakcqWGbbqqcqGGSaalcqWGwbGvcqGGPaqkcqGH9aqpdaaeqbqaamaaqafabaGaem4vaCLaeiikaGIaemyAaKMaeiilaWIaemOAaOMaeiykaKcaleaacqWGPbqAcqGHiiIZcqWGbbqqaeqaniabggHiLdaaleaacqWGQbGAaeqaniabggHiLdaaaa@4822@

Therefore the solution to the segmentation problem minimizes the affinity between groups normalized by the affinity within the same group. In this work, the spectral clustering is used for the first time for TRUS image segmentation using the approach proposed in [27].

## 4. Proposed lgorithm implementation

The segmentation proposed algorithm is composed of the following steps:

**1. The edge map **of the TRUS image is obtained using Canny edge detection method

**2. The Affinity matrix **is created using equation 3.

**3. The eigenvectors **are calculated and reshaped to be shown in the figures.

**4. Eigen vector Discretization**: In the best case scenario the second smallest eigenvector should take on two discrete values and the signs of the values can tell how to partition the graph. The second smallest eigenvector obtained in our case is a continuous vector; therefore it needs to be discretized in order to find a splitting point to obtain the clustering. In this work the splitting point that minimizes Ncut that is shown in equation (6) is chosen.

## 5. Validation methods

In the previous research that focuses on prostate boundary detection, several validation measures were used [14,16,29] such as:

***Distance δ ***= Average Euclidean distance (in pixels) between the algorithm-based segmentation and the manual segmentation. For each pixel the distance is defined as the shortest Euclidean distance between that pixel and the pixels located on the other contour.

***Area Difference AD : ***AD=100∗Smanual−SAlg⁡orithmSmanual
 MathType@MTEF@5@5@+=feaafiart1ev1aaatCvAUfKttLearuWrP9MDH5MBPbIqV92AaeXatLxBI9gBaebbnrfifHhDYfgasaacH8akY=wiFfYdH8Gipec8Eeeu0xXdbba9frFj0=OqFfea0dXdd9vqai=hGuQ8kuc9pgc9s8qqaq=dirpe0xb9q8qiLsFr0=vr0=vr0dc8meaabaqaciaacaGaaeqabaqabeGadaaakeaacqWGbbqqcqWGebarcqGH9aqpcqaIXaqmcqaIWaamcqaIWaamcqGHxiIkdaWcaaqaaiabdofatnaaBaaaleaacqWGTbqBcqWGHbqycqWGUbGBcqWG1bqDcqWGHbqycqWGSbaBaeqaaOGaeyOeI0Iaem4uam1aaSbaaSqaaiabdgeabjGbcYgaSjabcEgaNjabd+gaVjabdkhaYjabdMgaPjabdsha0jabdIgaOjabd2gaTbqabaaakeaacqWGtbWudaWgaaWcbaGaemyBa0MaemyyaeMaemOBa4MaemyDauNaemyyaeMaemiBaWgabeaaaaaaaa@552E@ where *S*_*Manual *_is the area of the manual segmentation and *S*_*Algorithm *_is the area of the algorithm-based segmentation.

***Area Overlap AO: ***AO=100∗Smanual AND SAlg⁡orithmSmanual OR SAlg⁡orithm
 MathType@MTEF@5@5@+=feaafiart1ev1aaatCvAUfKttLearuWrP9MDH5MBPbIqV92AaeXatLxBI9gBaebbnrfifHhDYfgasaacH8akY=wiFfYdH8Gipec8Eeeu0xXdbba9frFj0=OqFfea0dXdd9vqai=hGuQ8kuc9pgc9s8qqaq=dirpe0xb9q8qiLsFr0=vr0=vr0dc8meaabaqaciaacaGaaeqabaqabeGadaaakeaacqWGbbqqcqWGpbWtcqGH9aqpcqaIXaqmcqaIWaamcqaIWaamcqGHxiIkdaWcaaqaaiabdofatnaaBaaaleaacqWGTbqBcqWGHbqycqWGUbGBcqWG1bqDcqWGHbqycqWGSbaBaeqaaOGaeeiiaaIaemyqaeKaemOta4KaemiraqKaeeiiaaIaem4uam1aaSbaaSqaaiabdgeabjGbcYgaSjabcEgaNjabd+gaVjabdkhaYjabdMgaPjabdsha0jabdIgaOjabd2gaTbqabaaakeaacqWGtbWudaWgaaWcbaGaemyBa0MaemyyaeMaemOBa4MaemyDauNaemyyaeMaemiBaWgabeaakiabbccaGiabd+eapjabdkfasjabbccaGiabdofatnaaBaaaleaacqWGbbqqcyGGSbaBcqGGNbWzcqWGVbWBcqWGYbGCcqWGPbqAcqWG0baDcqWGObaAcqWGTbqBaeqaaaaaaaa@6A8C@

Since the main purpose of this paper is to introduce the new method and prove its concepts, one validation method is used which is the AO. The AO can be considered as a good representation for the algorithm success in segmenting the prostate as it measures the area of the gland that the algorithm could capture. The authors realize that more images are needed for the investigation to be generalized.

## 6. Experimental results

In this section, some results that show the correlation between the desired segmentation and the eigenvectors of the affinity matrix corresponding to TRUS images already segmented (either from the background or into cancerous and non cancerous regions) by an expert radiologist.

### 6.1. Segmenting the prostate from the background

**Figure 1:**
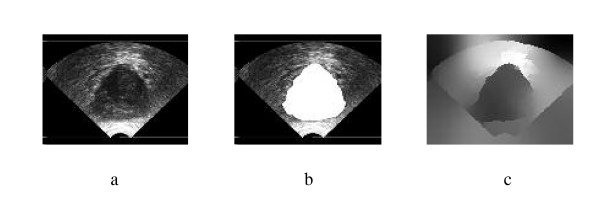
a) Original TRUS Image1, b) Manually Segmented, c)Second Eigenvector.

**Figure 2:**
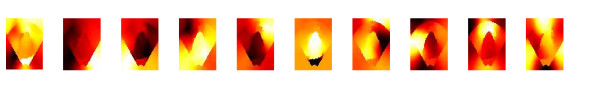
All Obtained Eigenvectors.

**Figure 3:**
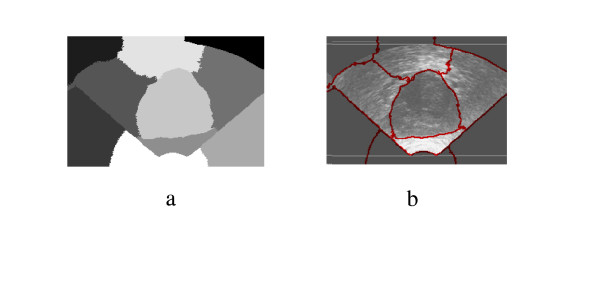
a) the obtained segmentation using the implemented algorithm, b) the contour map overlapped on the original TRUS image.

**Figure 4:**
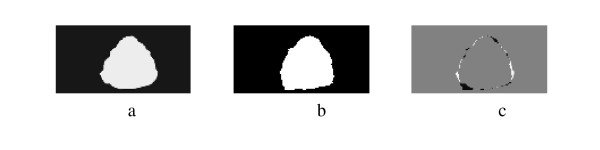
a) Manual Segmentation, b) Spectral Clustering Segmentation, c) overlap (92.86%).

Spectral Clustering was applied for medical image segmentation only in one recent publication [28]. Spectral Clustering was applied mainly to artificial ultrasound images and was then tested on a couple of ultrasound images obtained from vivo. Therefore more justification needs to be done to accept the algorithm results for prostate tissue classification and regions segmentation. In order to test the validity of applying the Spectral Clustering for the TRUS image segmentation for the purpose of tissue classification and to ensure that the internal segmentation of the gland is acceptable, the algorithm is being tested in this work with a problem whose solution is well defined. Therefore, Spectral Clustering is used to segment the prostate gland from the background and the results are compared to those obtained by the radiologist (considered better ground truth than recognizing the cancerous regions). Twenty nine Prostate images are segmented from the background using the proposed Spectral Clustering algorithm with high accuracy. The common areas between the doctor's segmented images and the spectral clustering segmented images are obtained and the average area for all images is 93%. The results are shown in the following figures where Figure 1 shows the original image, the radiologist's map and the second eigenvector reshaped to an image, which shows the high correlation between the radiologist mask and the second Eigen vector. Figure 2 shows all the obtained eigenvectors which show that the second eigenvector is the most correlated one with the desired segmentation. Figure 3 shows the proposed segmentation algorithm result. Figure 4 shows a comparison between the manually segmented prostate and the prostate segmented using the proposed algorithm.

**Figure 5:**
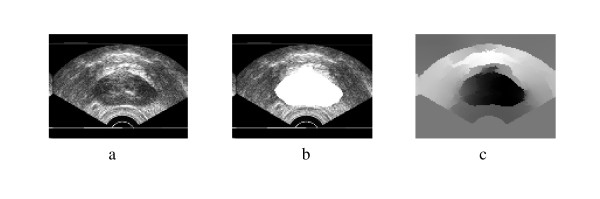
Original TRUS Image2, Manually Segmented, Second Eigenvector.

**Figure 6:**
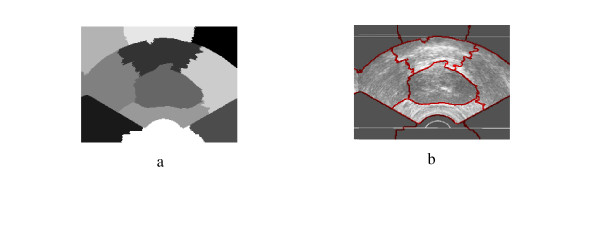
a) Segmentation Result, b) Obtained Contours.

**Figure 7:**
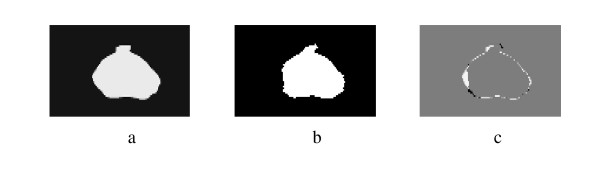
a) Manual Segmentation, b) Spectral Clustering Segmentation, c) Overlap (92.98%).

**Figure 8:**
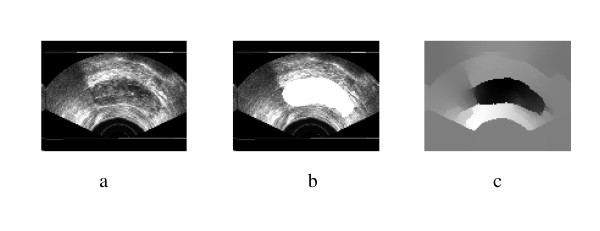
Original TRUS Image3, Manually Segmented, Second Eigenvector.

**Figure 9:**
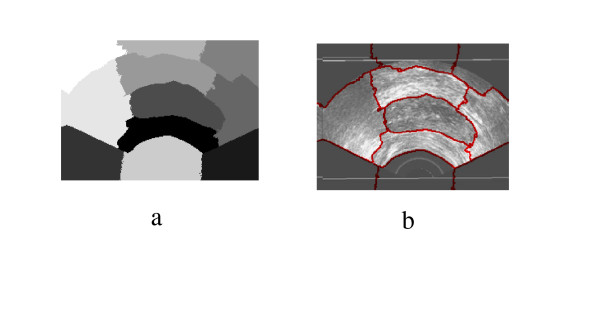
a) Segmentation Result, b) Obtained Contours.

**Figure 10:**
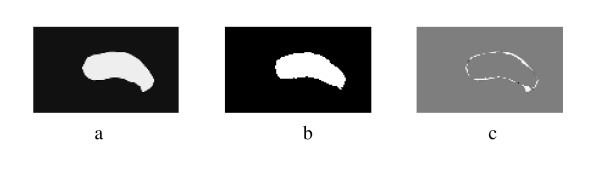
a) Manual Segmentation, b) Spectral Clustering Segmentation, c) Difference (93.03%).

More segmentation results are shown in Figure 5, Figure 6, Figure 7, Figure 8, Figure 9 and Figure 10. More images are shown in Additional file 1. The overlap areas in all the segmented images prove the high accuracy in segmenting the prostate from the background regardless of the prostate shape, location and orientation. Therefore the Spectral Clustering segmentation proved its excellent performance in segmenting the TRUS images. Therefore its results in segmenting the interior of the gland should be trusted as well.

### 6.2. Internal regions segmentation

The spectral clustering image segmentation algorithm is mainly used in this work for internal gland segmentation. However it was applied in the previous subsection for the prostate gland segmentation from the TRUS image and it obtained high segmentation accuracy which proves its capability of dealing with TRUS images effectively. Therefore, it is used in this sub-section for ROI (Region of Interest) segmentation from the manually segmented prostate.

The proposed algorithm in this work is faster than the Gabor multi-resolution analysis that was used earlier for prostate ROI identification [12] on the expense of giving a rough estimate of the internal segmentation than the earlier presented work. The algorithm proposed in this paper can then be used for suspicious regions estimation in an online application. It can also be used to support the decisions obtained using the feature analysis methods especially if the later contradicts the radiologist's decision.

#### Typical prostate shape

**Figure 11:**
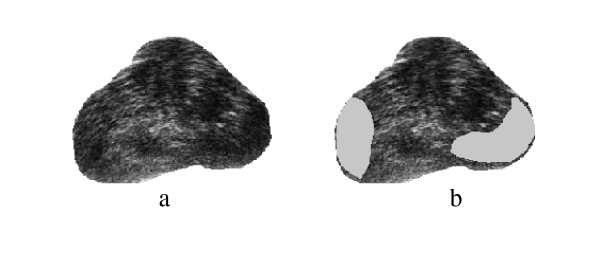
Prostate TRUS Image 10 with the desired segmentation.

**Figure 12:**
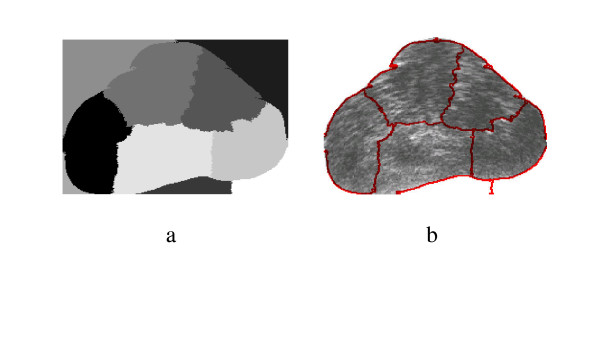
a) Segmentation Result, b) Obtained Contours.

**Figure 13:**
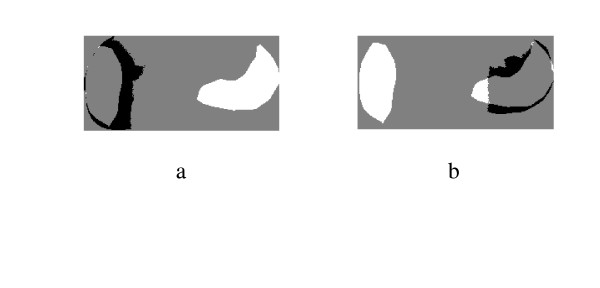
The Difference between each of the Identified regions and the Manual Segmentation.

The proposed spectral algorithm was successful for capturing the suspicious cancerous regions from the TRUS images with typical prostate shape. The original gland, the corresponding doctor's segmentation and the corresponding regions contours are shown in Figure 11, 12 and 13, and another example is shown in 14, 15 and 16. The results show that the algorithm was successful in identifying the doctor's suspicious regions in the typical gland. The proposed Spectral Clustering algorithm did capture the information that was hidden from the radiologist's decision and can't be seen by the naked eye which confirms and supports the decision of the recognition methods explained earlier in the thesis. Therefore, it can be concluded that the Spectral Clustering algorithm recognized regions that were missed by the radiologist, yet still carry the same information of the radiologist identified regions which proves the superiority of the proposed algorithm.

**Figure 14:**
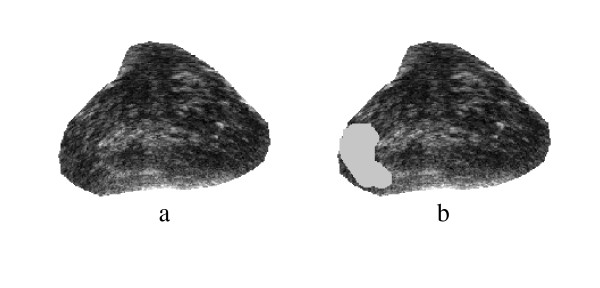
Prostate TRUS Image 11 with the desired segmentation.

**Figure 15:**
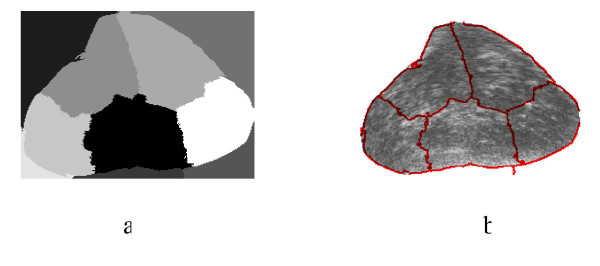
a) Segmentation Result, b) Obtained Contours.

**Figure 16:**
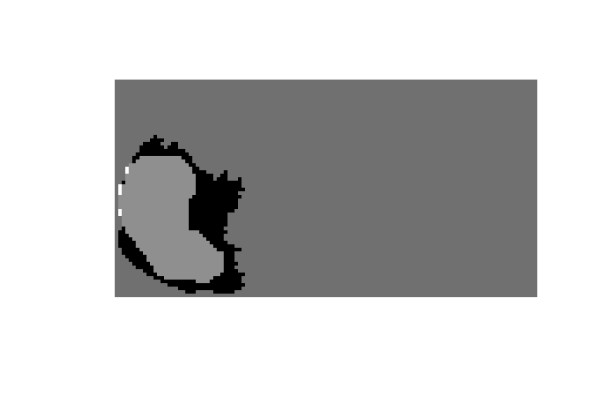
The Difference between the Identified region and the Manual Segmentation.

#### Unusual prostate shape

The spectral clustering algorithm also shows excellent segmentation even if the prostate shape is not the typical shape. Another example of unusual prostate shapes is shown 17, 18 and 19. The last sample image and its corresponding segmentation is shown in 20, 21 and 22. The proposed segmentation algorithm did capture a rough estimate of the suspicious regions and their location in the gland even in the atypical gland shape which is considered confusing for the registration algorithms. This proves the ability of the algorithm to be used as a preliminary online estimate for the cancerous regions as well as a support for the existing CAD methods that involve ROI identification.

**Figure 17:**
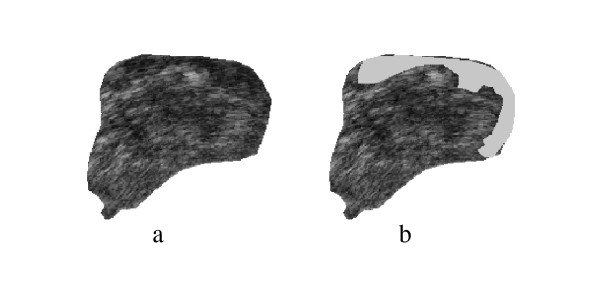
Prostate TRUS Image 12 with the Desired Segmentation.

**Figure 18:**
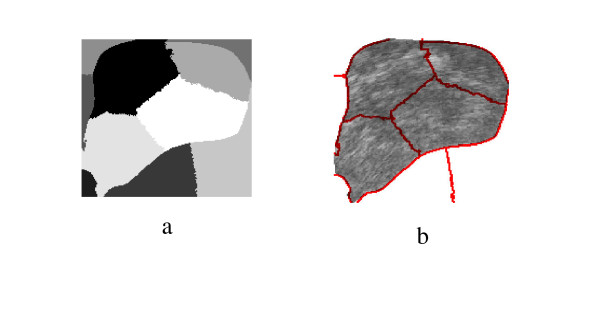
a) Segmentation Result, b) Obtained Contours.

**Figure 19:**
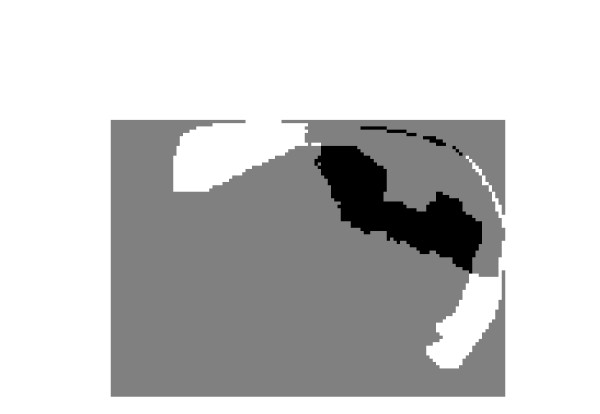
The Difference between the Identified region and the Manual Segmentation.

**Figure 20:**
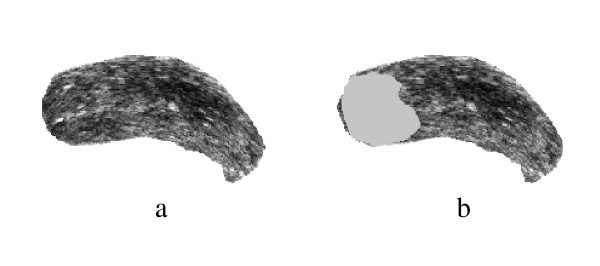
Prostate TRUS Image 13 with the Desired Segmentation.

**Figure 21:**
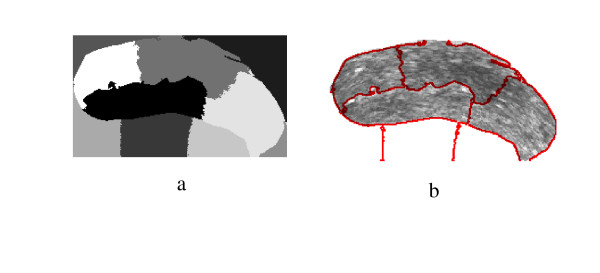
a) Segmentation Result, b) Obtained Contours.

**Figure 22:**
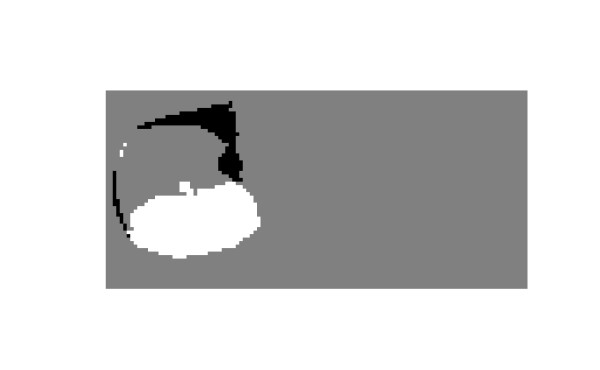
The Difference between the Identified region and the Manual Segmentation.

## 7. Discussion

From the above results it is clear that the spectral clustering can be used as a preliminary estimate for the cancerous regions during the imaging procedure. Moreover it can be used as a support for the decision making either by the radiologist or by the other CAD systems. It can also estimate the prostate volume, location and size from the TRUS image effectively. The second smallest eigenvector proved to be consistent with the radiologist manual segmentation for the two different segmentation problems tackled in this paper

## 8. Conclusion

In this paper a novel technique is proposed to segment the prostate using TRUS images. The new strategy that is introduced in this work is based on the spectral clustering algorithm. Spectral clustering has the benefit of being built on a totally different foundation that doesn't include any contour or seed point estimation. The proposed spectral clustering segmentation method is inspired from the graph theory techniques. The idea of the spectral clustering depends mainly on treating the image as a weighted graph and searched for the minimum cut of that graph. This idea has a totally different prospective than the well known prostate segmentation methods such as the snakes. The proposed spectral clustering segmentation method is accurate, simple to implement and doesn't involve any energy functions to be built or optimized. From the results and analysis shown in this paper, it can be concluded that spectral clustering can be considered as a new advance for prostate segmentation that proved its ability to accurately segment the gland from the background for typical as well as atypical prostate shapes. Moreover, it is also clear from the results obtained in this work that the spectral clustering method is able to roughly segment the cancerous regions that proved consistency with the regions identified by the doctor. The proposed method is able to recognize regions regardless of the prostate shape and the spatial location of the cancer within the gland.

In conclusion, the algorithm obtained fast excellent estimates that can give rough prostate volume as well as cancerous regions segmentation which can be used for online application.

## Supplementary Material

Additional file 1 Appendix 1. The file contains some of the resulting segmented prostate glands from the TRUS images as well as their manually segmented counterparts. Click here for file
