# Supplementary material for: Spectral clustering for TRUS images
Source: Biomed Eng Online. 2007 Mar 15;6:10. doi: 10.1186/1475-925X-6-10 (PMC1845149; doi:10.1186/1475-925X-6-10)
Supplement: Additional file 1 — Appendix 1. The file contains some of the resulting segmented prostate glands from the TRUS images as well as their manually segmented counterparts. [file 1475-925X-6-10-S1.doc]

**Appendix**

Following are some of the segmented prostate glands from the TRUS images. Where the image to the left of each figure shows the radiologist segmented gland. The image in the middle shows the Spectral Clustering segmented gland and the image to the right of each figure shows the overlap image that illustrates the common areas between both the radiologist segmented and the spectral clustering segmented images.
